# Supplementary material for: Extracellular Polysaccharide Receptor and Receptor-Binding Proteins of the Rhodobacter capsulatus Bacteriophage-like Gene Transfer Agent RcGTA
Source: Genes (Basel). 2023 May 22;14(5):1124. doi: 10.3390/genes14051124 (PMC10218006; doi:10.3390/genes14051124)
Supplement: Supplementary file 1 [file genes-14-01124-s001.zip › genes-2394956-supplementary.pdf]

Table S1. E-values of top hits in BLASTp analysis of the 37b4 genome using WT strain SB1003 homologues of cosmid pCPS1-encoded protein sequences as queries, and reciprocal BLASTp hits.

SB1003 query locus/gene name	37b4 top hit locus/gene name (E-value)	reciprocal blast hit SB1003 locusgene name (E-value)
rcc01067	none	N/A
rcc01068	none	N/A
rcc01069	none	N/A
rcc01070	none	N/A
rcc01071	none	N/A
rcc01072/*hutH*	none	N/A
rcc01073/*gvpA*	none	N/A
rcc01074	none	N/A
rcc01075	*rcc01834*/*mcpH* (5E-76)	*rcc01726*/*mcpH* (0)
rcc01076	none	N/A
rcc01077	*rcc01848* (2E-48)	*rcc01077* (6E-61)
rcc01078	*rcc01849* (5E-50)	*rcc01078* (2e-44)
rcc01087/*pip2*	*rcc01521*/*pip* (0.001)	*rcc02274*/*pip* (0)
rcc01088	*rcc02269* (9E-09)	*rcc01508* (4e-145)
rcc01079	*rcc02299* (5E-05)	*rcc00962* (3E-41)
rcc01080	none	N/A
rcc01081	*rcc01327* (2E-04)	none
rcc01082	none	N/A
rcc01083/*lspl1*	*rcc02081* (0)<br>*rcc00179* (5E-29)	*rcc01952*/*lspl2* (0)
rcc01084	none	N/A
rcc01085	none	N/A
rcc01086	*rcc03284* (0)	*rcc03089* (0)
rcc01932	*rcc02060* (E-174)	*rcc01932* (0)
rcc01958/*wzc*	*rcc02087* (0)	*rcc01958*/*wzc* (0)
rcc01959/*wzb*	*rcc02088* (2E-83)	*rcc01959*/*wzb* (0)
rcc01960/*wza*	*rcc02089* (0)	*rcc01960*/*wza* (0)
rcc00197/*comF*	*rcc00201* (5E-132)	*rcc00197*/*comF* (3E-122)
rcc00460/*comM*	*rcc00465* (0)	*rcc00460*/*comM* (0)
rcc03098/*dprA*	*rcc03293* (0)	*rcc03098*/*dprA* (8E-134)
rcc01751/*recA*	*rcc02029* (0)	*rcc01751*/*recA* (0)
rcc00222/*radC*	*rcc00227* (0)	*rcc00222*/*radC* (0)

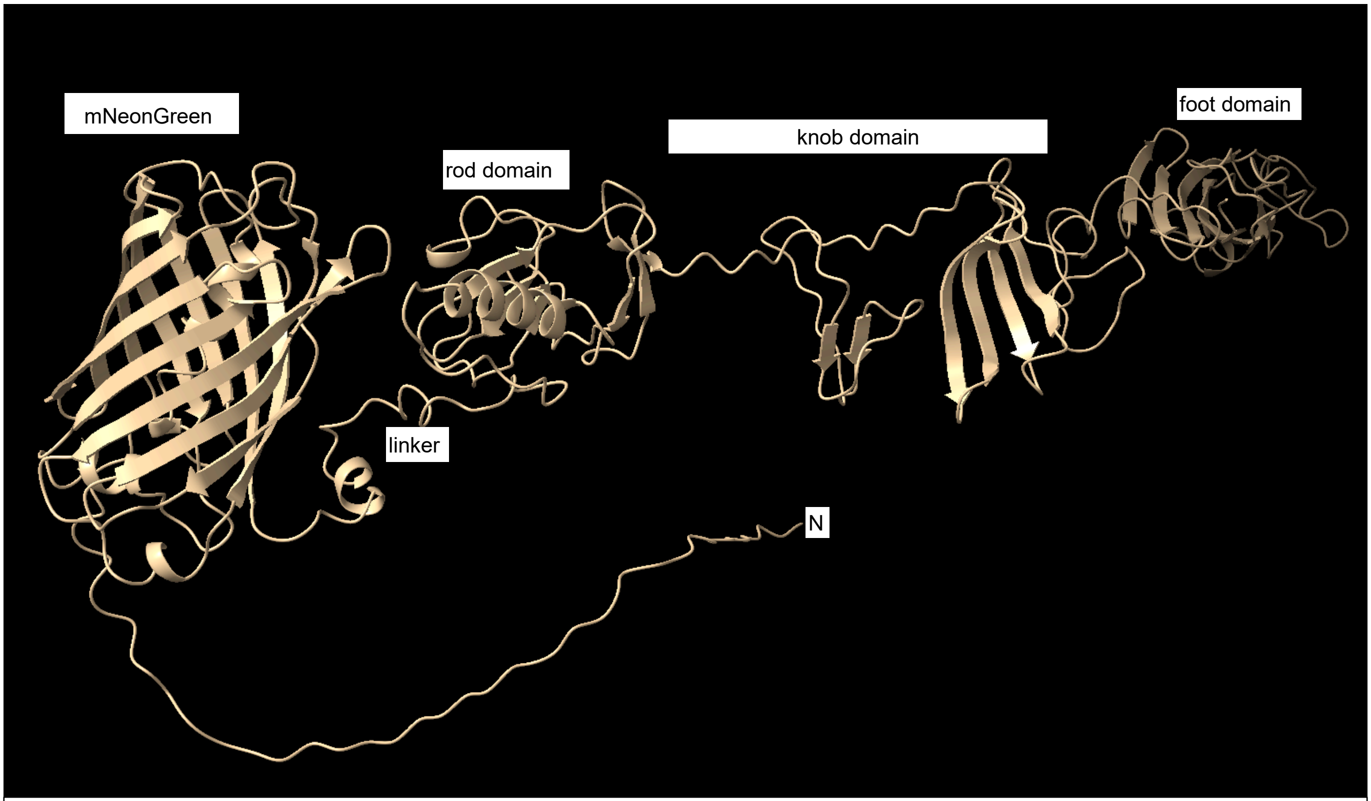

Figure S1. AlphaFold predicted structure of mNeonGreen fluorescent protein fused to the N terminus of the RcGTA tail fiber.

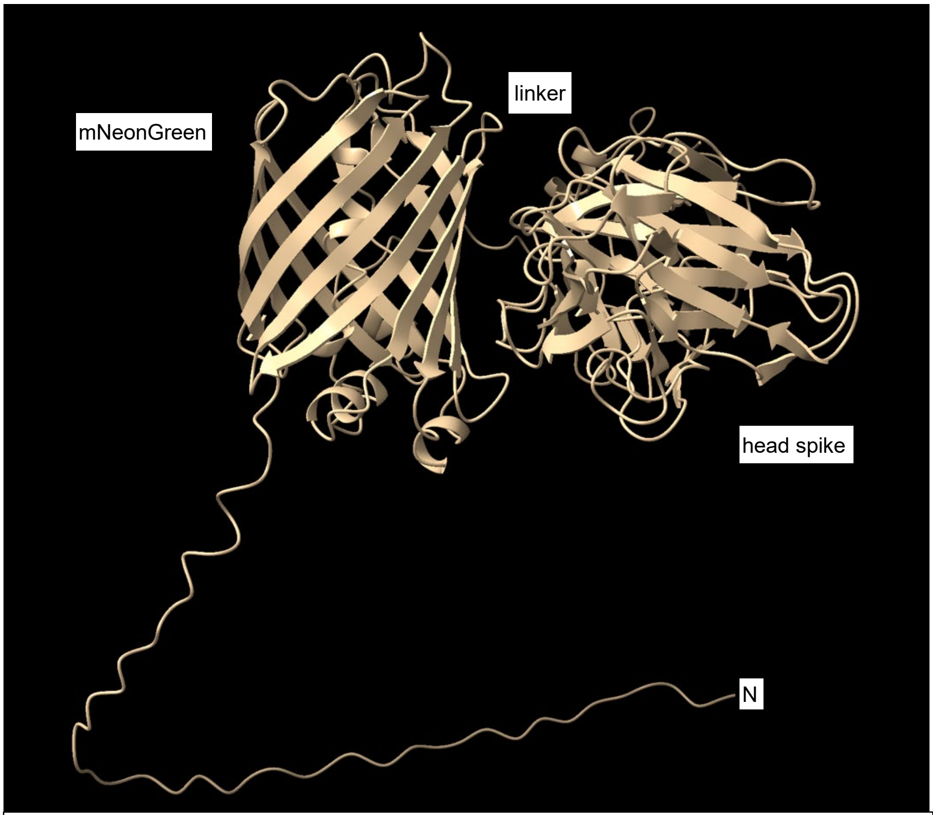

Figure S2. AlphaFold predicted structure of mNeonGreen fluorescent protein fused to the N terminus of the RcGTA head spike fiber.
